# The Impact of the Fresh Pork Display Lamps on the Sensory Response of Consumers to Fresh Pork

**DOI:** 10.3390/foods13121827

**Published:** 2024-06-11

**Authors:** Yixuan Liu, Wei Chen, Xinwei Wu, Michael Pointer, Zhengjie Chen, Xinghai Liu, Qiang Liu, Xufen Xie

**Affiliations:** 1 Department of Psychology, Academy of Advanced Interdisciplinary Studies, Wuhan University, Wuhan 430072, China; yxl2021@whu.edu.cn (Y.L.); chenwei1011@whu.edu.cn (W.C.); xinwei.wu@whu.edu.cn (X.W.); 2017301750025@whu.edu.cn (Z.C.); liuxh@whu.edu.cn (X.L.); 2 School of Design, Colour Technology Research Group, University of Leeds, Leeds LS2 9JT, UK; mrpointer@btinternet.com; 3 Joint Laboratory of Light Quality and Colour Vision, Wuhan University & OPPLE, Wuhan 430072, China; 4 School of Information Science and Engineering, Dalian Polytechnic University, Dalian 116034, China

**Keywords:** fresh pork display lamp, pork products, freshness evaluation, freshness distinction, purchase experience

## Abstract

Two studies were conducted to investigate the impact of fresh pork display lamps on consumers’ sensory responses to pork products. In the first experiment, 63 participants were asked to evaluate pork products with different degrees of freshness under four fresh pork display lights and two LED lights. In the other experiment, 30 subjects participated in the Farnsworth–Munsell 100 hue test under the same lamps, with the aim of showing whether the fresh pork display lamps impaired color discrimination. The results showed that the light source had a significant effect on the color appearance evaluation of the pork products. The ratings for perceived freshness under the fresh pork display lamps were significantly higher than those of ordinary LED lamps, while the color discrimination performance of the subjects under those lamps was poor. It was demonstrated that improper component proportions of the light spectrum could influence observers’ assessment of meat quality and weaken the observers’ ability to distinguish the freshness level. Through this study, the authors would like to remind lamp users and manufacturers to not only be concerned about the improvement in the color appearance of pork, but also the need for consumers to be aware of the authentic freshness of the pork products.

## 1. Introduction

With the improvement in the social economy as well as consumers’ quality of life, people’s eating patterns have changed. In the past decades, along with the rapid increase in meat consumption, the proportion of animal protein in the total protein intake of consumers has significantly increased. According to the predictions of the Organization for Economic Cooperation and Development (OECD) and Food and Agriculture Organization (FAO) of the United Nations, growth in the global consumption of meat proteins over the next decade is projected to increase by 14% by 2030 compared to the base period average of 2018–2020, driven largely by income and population growth. Global pigmeat consumption is projected to increase to 127 Mt over the next ten years and to account for 33% of the total increase in meat consumption. China is projected to account for most of the total increase in meat production and will account for 70% of the increase in pigmeat consumption from the reference period to 2030 [1].

In recent years, food safety has aroused worldwide attention and become one of the most important global issues. The improper handling of food during production, distribution, preparation, and sale can lead to serious public health risks. The World Health Organization concluded that globally almost 1 in 10 people have become ill after eating contaminated food and 420,000 have died, resulting in a loss of 33 million healthy life years [2]. Thus, as one of the most important contributors to a meat diet, the quality of pork products is fully valued by both suppliers and consumers.

In the domain of sensory analysis, the human-based assessment usually includes the analysis of various attributes, such as appearance (size, color, shape, visual defects, etc.), texture, smell, and flavor. Among the various attributes, surface color is usually considered as the primary characteristic for evaluating the food quality [3,4,5]. This is particularly true for meat products since they are primarily assessed based on visual appearance rather than packaging. Visual perception is often the first organoleptic attribute that elicits arousal and appetite in humans when ultimately deciding whether to choose or reject a food [6,7]; therefore, the meat color preference of consumers was identified as a predictor of purchasing intent [8].

The importance of meat color was demonstrated by Naumann et al. [9], who announced that consumers consider two aspects in meat purchasing: one is visual appearance while the other is palatability, which is determined by the overall quality of the meat. Obviously, it is not possible for consumers to evaluate the flavor, tenderness, and juiciness of meat in a retail display, so they have to make their selection depending on the evaluation of the visual appearance. As Pangborn [10] stated, “to a large extent, man recognizes, discriminates and selects nutrients with the eye. Through conditioning and association, he expects an item of certain shape and colour to have a specific odor, taste and texture.” Color, therefore, is the key parameter that influences consumer choice at the point of purchase [11,12,13,14].

Meat showing a bright red color is assumed to be fresh, while the oxidation of heme iron and the form of the metmyoglobin (MetMb) produce an undesirable brown color [15]. In fact, meat color is affected by numerous factors. The internal factors are breed, slaughter, meat ripening, feeding, water retention, and type of muscle tissue [16], while the external factors include storage time, atmosphere, light source, and light intensity [17,18,19]. Theoretically, color is generated by the interaction between the object, the eye, and the light source and we also know that meat quality is influenced by light deterioration [20,21,22]. Thus, the light source can be extremely important for the display and storage of meat products.

Among previous studies, the color stability during meat storage has been extensively explored. Martínez et al. [15] demonstrated that UV radiation was highly deleterious for the display life of frozen pork sausages. Similar results were reported by Djenane et al. [23] for frozen beef steaks. Steele et al. [24] indicated that LED lighting in fresh meat retail displays offers benefits in extending the color shelf life of pork loin chops as well as other kinds of meat.

On the other hand, the impact of light on the sensory evaluation of meat appearance and freshness has also been discussed. Clark [25] reported that light sources with a closer fit to the reflectance pattern for any product or muscle will bring out the naturally appetizing appearance of that product, so they recommended that meat light sources should be reasonably rich in the red part of the spectrum. Hunt et al. [26] announced that an incandescent light source may mask the color deterioration of frozen lamb chops compared to a deluxe cool white, fluorescent lamp. Barbut [27] demonstrated that the color of fresh meat is seen as more desirable under incandescent lamps compared to fluorescent and halogen lamps and also found that the color of pork chops was more preferred under high-spectrum fluorescent lamps than under incandescent or Chroma 50 fluorescent lamps [28]. These studies pay more attention to the instant sensory response of human observers on the color appearance and perceived freshness of meat products.

In recent years, a large number of specific lamps that can be used to illuminate fresh food products have emerged on the market. For example, in a study of Smet et al. [29], the authors used two lamps, named Red Premium and Rosé, which are meat lighting specialty products from Signify (formerly known as Philips Lighting) and two other common lamp spectra which are also used in meat lighting to carry out the color quality evaluation. According to the types of illuminated objects, fresh food display lamps can be classified into many categories such as display lamps for pork, beef, seafood, fruit and vegetables, etc. Unlike the traditional light sources used in past studies, these lamps generated light with certain combinations of narrow band spectra (e.g., red and blue, yellow and white, green and white) and produced much more conspicuous visual effects. In the past few years, these lamps were common in fresh meat counters, supermarkets, delis, fruit and vegetable shops, and seafood markets, especially in China. Among these specific lamps, those used to illuminate pork were known as fresh pork display lamps. They were designed to promote the consumers’ evaluation of the meat freshness by beautifying the color appearance of meat without changing its real properties. At the same time as the introduction of these specific lamps, consumer complaints about pork quality have increased. Purchasers have usually complained that under these special light sources with very strong reddish and bluish emissions, pork products look tender and glossy, but when viewed in natural daylight or normal white light, the pork products look much less colorful and even stale. Meanwhile, another complaint from consumers was that they cannot distinguish colors under these fresh pork display lamps. Similar problems have also been reported by Mahler et al. [30], who found that the discrimination efficiency of illumination is reduced due to the falsely saturated colors. Therefore, since December 2023, the usage of such light sources has been banned in China, but they are still in use in some other countries like Australia and Korea.

Thus, in this study, we investigated this topic by sensory studies. To our knowledge, no past work has been performed exploring the impact of the fresh pork display lamps on the evaluation of pork freshness. So, we aimed to explore the impact of the fresh pork display lamps on people’s color perception and visual evaluation of the freshness of fresh pork products. In addition, due to the complaints that the fresh pork display lamps deteriorate the color discrimination ability of consumers, that particular visual attribute of the lighting was also investigated using a sub-test of the Farnsworth–Munsell 100 hue test (FM-100) [31] to check the color discrimination under these special lights. In addition, inspired by the work of Wang et al. [32] that highlighted the impact of experience on purchasing willingness for red meat, the impact of purchasing experience on pork appearance evaluation was also considered by inviting different groups of subjects with and without pork purchasing experience to participate in this study. 

It is hoped that the research findings will provide a deeper understanding of fresh pork display lighting, especially for consumers and market sellers, as well as fresh pork display lamp manufacturers who constitute part of the sales chain of pork products. 

## 2. Materials and Methods 

### 2.1. Sensory Evaluation

#### 2.1.1. Participants

The experiments described here were approved by the Ethics Committee of the research center of graphic communication, printing, and packaging, Wuhan University. The project identification code is 2172021011 (approved on 20 October 2021). All subjects provided written informed consent in accordance with the Declaration of Helsinki. The subjects were informed about the testing procedure before the test and about the aims of the experiment after the test with the option of deleting their data, if then they did not wish to participate in the test. Participants were able to withdraw from the experiment at any time without stating a reason and they were financially compensated for their participation in the amount of CNY 30.

To consider the impact of purchasing experience, we interviewed 151 people before the experiment. Through the investigation of the frequency of buying meat, we selected two groups of observers who met the purpose of the experiment. We classified those who bought pork more than six times a month as experienced participants, and those who seldom bought pork as inexperienced. Finally, 63 volunteers participated in the evaluation experiment, including 32 people (10 males and 22 females) with no experience in buying pork products and 31 people (3 males and 28 females) who regularly bought pork products. Among them, most of the inexperienced participants were young people, while the experienced were mostly middle-aged. The average age of the inexperienced group was 22.4 years (SD = 1.3), while the average age of the experienced group was 48.3 years (SD = 2.2). All participants either worked or studied at Wuhan University and had passed the Ishihara Color Vision Test. 

#### 2.1.2. Experimental Light Sources

**Figure 1:**
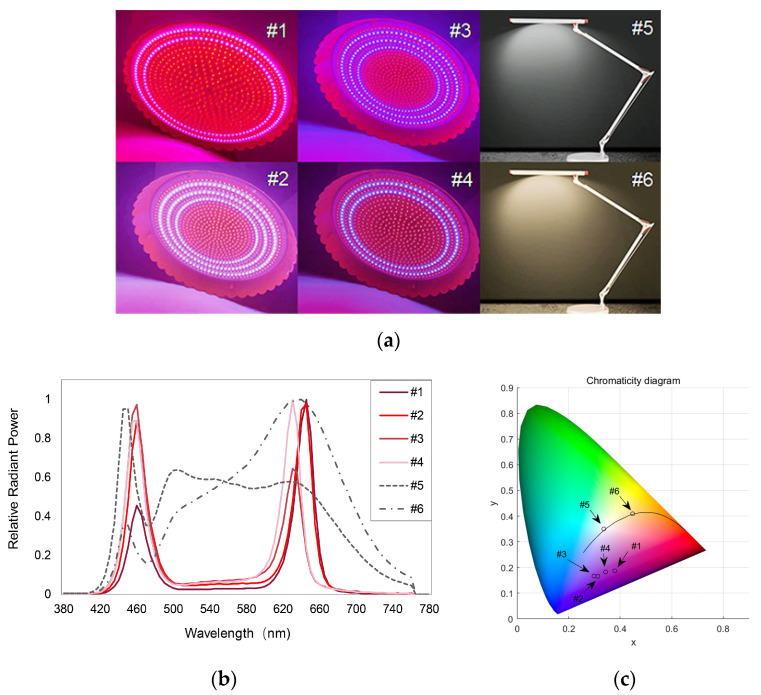
(**a**) The six lamps used in the experiment; (**b**) relative spectral power distributions of the light sources; (**c**) CIE x, y chromaticity coordinates of the light sources (color online only).

The experiment was conducted in a dark room at Wuhan University, which was furnished with 4 fresh pork display lamps and 2 standard LED lamps with correlated color temperatures (CCTs) of 5356 K and 2861 K, respectively. The lamps used in the experiment, as well as their spectral power distributions (SPDs) and chromaticity, are illustrated in Figure 1. The fresh pork display lamps (#1 to #4) were purchased from a local lighting market and each contained different white and red LEDs. The two standard LED light sources (#5, #6), which were used as the control lamps, were generated by a color-tunable desk lamp. The illuminance levels of the six light sources were set to be 1500 lx, according to the results of a preliminary survey of 16 meat counters in Wuhan, China. An Everfine SPIC-300AW spectral irradiance meter (EVERFINE, Hangzhou, China) was used to measure the relative SPDs, shown in Figure 1. The colorimetric properties of the light sources together with typical color quality measures are summarized in Table 1. 

#### 2.1.3. Meat Samples

**Figure 2:**
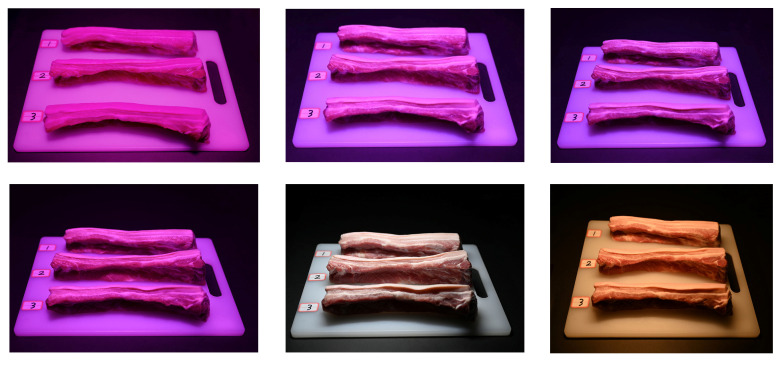
Overview of the experimental setup. From left to right and top to bottom, the pork cuts are captured under fresh pork display lamps #1, #2, #3, #4, and standard LED lamps #5 and #6 (color online only).

Pork belly joints with three degrees of freshness were used as the objects for observation. The meat samples were carefully selected to ensure that they were as consistent as possible in size, weight, shape, color, and lean meat content, as shown in Figure 2. The first two groups of pork joints were purchased two days and one day before the experiment and then stored in the refrigerator at 4 °C for 48 h and 24 h, respectively, inside the packaging of polyethylene plastic bags that were used for packing food. A third group of pork joints was purchased on the day of the experiment and was also packed inside the polyethylene plastic bags before the experiment started on that day. These three groups of meat were all purchased from a local fresh supermarket in Wuhan, with the same origin and slaughtering process. To avoid observers identifying the pork cut by the lean–fat texture during the sensory experiment and thus making the same judgment under all lamps, 6 cuts of pork belly joint were prepared for a defined group of meat with a certain degree of freshness. Thus, 18 pork cuts were prepared for a one-day trial and 36 pork cuts were prepared in total for the whole experiment.

During the experiment, three pork cuts, each randomly selected from one group of freshness, were simultaneously displayed under one of the six experimental light sources, as shown in Figure 2. A white tray (40 cm × 30 cm) was used to hold the meat cuts and was placed on a black tablecloth against the grey wall. The position numbers (i.e., 1, 2, 3) on the tray were used to help the subjects to rate the freshness of the pork while the positions of the pork cuts with different degree of freshness were randomized and counterbalanced during the whole test. 

To quantify the degree of pork freshness, before the visual experiments, the TVB-N contents were determined by steam distillation according to the method described by the National Standard of the People’s Republic of China [38]. TVB-N is widely considered as an effective indicator of meat deterioration, since it has positive association with both microbiological proliferation and the activities of proteolytic enzyme—both major mechanisms for spoilage [39]. The results showed that the TVB-N values of the three groups of pork (i.e., newly purchased, refrigerated for 24 h after purchase, and refrigerated for 48 h after purchase) were 4.0 to 4.6 mg/100 g, 6.2 to 6.9 mg/100 g, and 9.3 to 9.9 mg/100 g, respectively. Such results indicate that all the meat products used in the experiments met the national standard of China which requests that the TVB-N value of fresh pork should be no larger than 15 mg/100 g [40]. In addition, it is clearly demonstrated that the three groups of pork cuts examined in the study were of distinct difference in freshness. 

**Figure 3:**
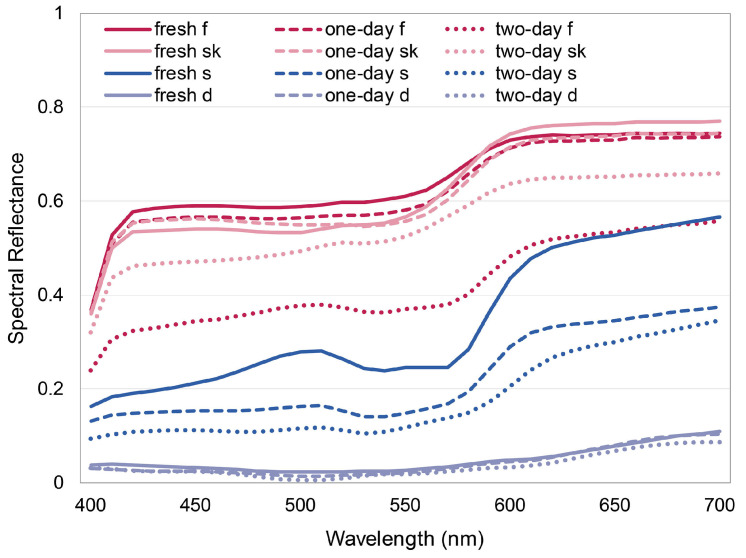
Spectral reflectance of typical colors of the experimental pork. “d”, “s”, “f”, and “sk” indicate a deep portion of the lean meat (darker), a superficial portion (paler), fat, and pigskin, respectively. “fresh”, “one-day”, and “two-day” indicate pork products purchased on the day of the experiment, and purchased one day and two days before the experiment, respectively (color online only).

Figure 3 illustrates the spectral reflectance of typical colors in the experimental pork product (i.e., pigskin, fat, superficial, and deep portion of lean meat), which was acquired before observation, using a spectral imaging system developed in our lab [41]. The 140 color patches in ColorChecker Digital SG charts were used as training samples. The spectral reflectance of the sample set was measured with an X-rite SpectroEye spectrophotometer. The samples and pork were placed horizontally in the center of a light booth and captured with a Canon 600D trichromatic digital camera at an angle of 45°. A stable light source was generated by an LED-cube spectrally tunable smart lighting system (Changzhou Thouslite Ltd.) with a CCT 5000 K and an illuminance level of 600 lx. The parameter settings for the camera were fixed during capture, with an aperture of f/5.0, an exposure time of 1/25 s, and an ISO rating of 400. Based on the camera RGB responses and the spectral reflectance data of the training samples, the spectral reflectance of the pork products was reconstructed, as shown in Figure 3. Note that in Figure 3, each measurement is the mean of five non-overlapping readings. There were twenty measurements taken in each pork cut sample (five measurements × four parts). The twenty measurements were made in each of the thirty-six pork cut samples.

#### 2.1.4. Experimental Design

**Figure 4:**
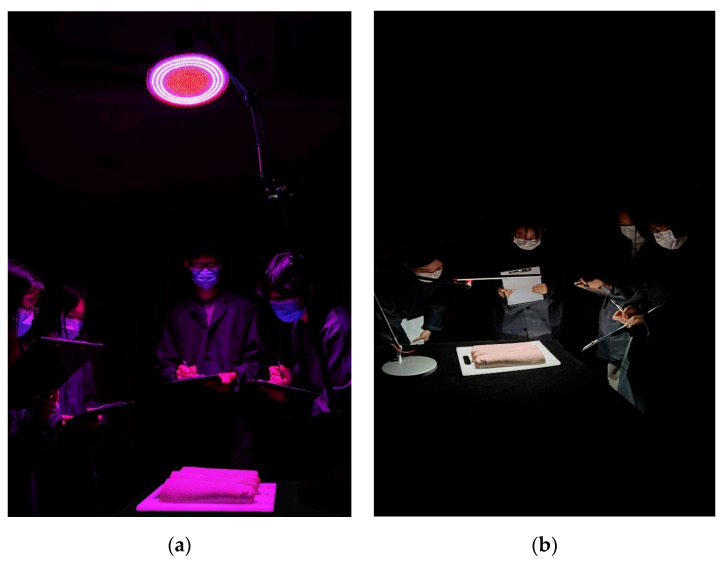
The experimental scene of the sensory evaluation experiment. (**a**) Under fresh pork display lamp; (**b**) under color-tunable desk lamp (color online only).

The tests were conducted in sixteen groups of four or three participants on two consecutive days. In each set of experiments, the distribution of two observer groups was balanced. For two different experimental days and different test trials in each day, there were both experienced and inexperienced participants to observe the same meats in that test trial. The pork samples were displayed under the lamps at a room temperature approximately 16 °C. The participants were instructed to stand next to each other and observe the samples under a lamp and they were not allowed to touch or smell the meat during the test (Figure 4). They were asked to respond with their ratings on *visual preference, freshness*, and *purchase intention* according to the appearance of meat using a 7-point rating method, ranging from 1 (not at all) to 7 (very). Participants were asked not to communicate with each other during the test. The order of the experimental light sources was randomized within each test and between tests. During each test, a randomly selected light source was rated twice to help in quantifying the intra-observer variability of each observer. It should be noted that these two repeated evaluations were not consecutive but arranged in a random order between all seven test lights (6 lamps + 1 repeated lamps). Therefore, in the experiment, each observer made a total of 63 evaluations as follows: 7 light sources (6 lamps + 1 repeated trial) × 3 meat samples × 3 visual responses. Each group of observers took about 35–40 min to complete the experiment.

#### 2.1.5. Experimental Procedure

Upon arrival, the participants were instructed to sign a consent form and were screened privately for color vision deficiencies using an Ishihara Color Vision Test. Then, the participants were asked to fill in their name, sex, age, and frequency of pork purchase on the questionnaire. The experimenter then asked the observers to wear grey coats so that there would be no color light reflected from their clothes onto the test samples. We also asked observers to wear masks to ensure that their judgments were not affected by odour. Subsequently, the ambient lights were switched off so that the experimental lighting was the only illumination in the room.

Prior to the start of the experiment, each observer was allowed approximately 3 min to adapt to the otherwise dark room, with only one lamp switched on. During that time, the experimenter read the instructions to the observer. At the beginning of the formal experiment, the experimenter asked the participants to close their eyes and changed the light source to one which was randomly chosen from the six experimental light sources. This step lasted for 20 s and was repeated every time the experimental light was changed, with the aim of eliminating the short-term memory effect of the former lighting. The experimenter then asked the participants to observe an A2 piece of neutral grey paper (420 mm × 594 mm) for 2 min to achieve sufficient chromatic adaptation. After adapting to the light, the participants were asked to close their eyes again. Then, the experimenter put the tray and pork on the table and asked the participants to observe the appearance of the meat samples and record their responses on the rating sheet provided. During the observation, the experimenter reminded the observers to move around the lamp so that they could obtain a full view of the pork; such a protocol aimed to simulate a real meat shopping scenario. After all participants had completed evaluating the pork samples under one light source, they were asked to close their eyes. At that time, the experimenter turned off the former light and switched on another lamp and then invited observers to step toward that lamp for assessment. Such a procedure was repeated for all the experimental light sources with a randomized and counterbalanced order, including the repeated light for quantifying intra-observer variability. Throughout the experiment, we also reminded participants not to look directly at the light sources and to focus only on the color appearance of the pork in front of them. After all the tests, we conducted a short interview with the participants about the judging criteria they used for rating the pork cuts.

### 2.2. Color Discrimination 

**Figure 5:**
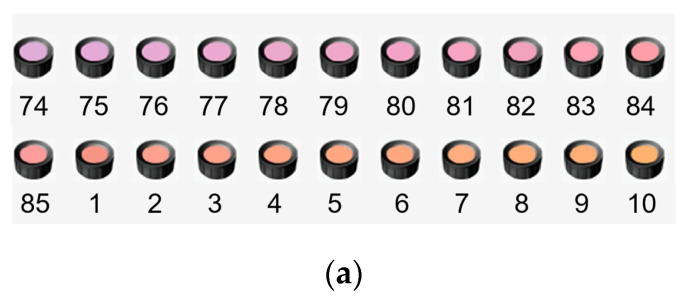

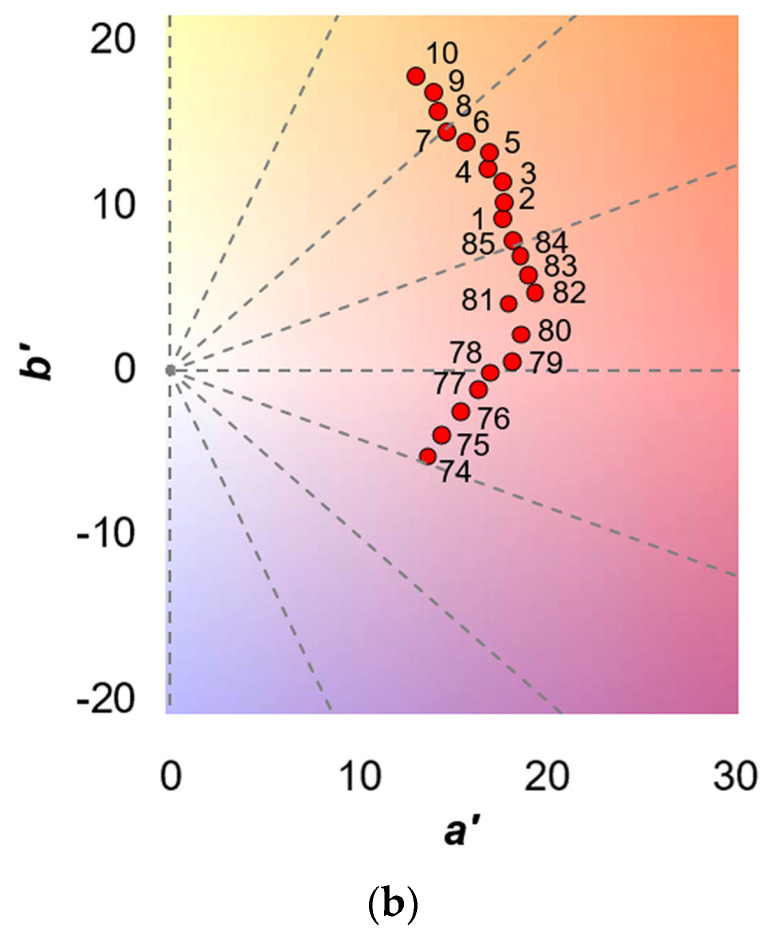
(**a**) The twenty-two color caps in the FM−100 hue test; (**b**) the corresponding hue distribution in the a’ b’ plane of CIE CAM02-UCS [43] (color online only).

The color discrimination capability of light source refers to “a measure of the extent to which the illumination allows the observer to discriminate among a large variety of object colours simultaneously viewed” [42]. To examine this capability of the experimental light sources, we designed a sub-test based on the FM-100 hue test. As shown in Figure 5a, twenty-two color caps from the red region in the FM-100 hue test were selected, which included 11 samples (74~84) from the purple–red tray and 11 samples (85~1~10) from the red–green tray. These samples were approximately consistent in lightness and chroma but exhibited perceptually uniform hue steps and they completely covered the hue ranges of pork colors. The corresponding hue distribution of these colors is shown in Figure 5b. For each cap, an integer number is provided on its underside to identify its position in the hue circle. During the test, observers were requested to rearrange the disordered 22 color caps into a continuous hue order.

The procedure and protocol of the color discrimination test were very similar to the earlier sensory evaluation test, with the same light sources and experimental settings. Thirty observers (AVG = 47.5 years, SD = 1.6) with normal color vision and pork purchasing experience were recruited. Among them, there were six males and twenty-four females. The ethical statements were the same as for the sensory evaluation experiment. The order of the experimental light sources was also randomized within each test and was different between tests. The time for each subject to finish each trial under each experimental light was recorded. On average, it took approximately 25–30 min for each participant to finish the test.

### 2.3. Data Analysis

For the sensory evaluation experiment, we calculated the standardized residual sum of squares (STRESS) [44] values to quantify the intra- and inter-observer variability. To quantify the intra-observer variability, the observers were required to rate a randomly selected lighting condition twice without being informed of this, then the STRESS values between the two ratings were calculated. To assess the inter-observer variability, the STRESS values were calculated between each observer’s ratings and those of the average observer. 

The data obtained from the participants in the two experiments were then analyzed using the IBM SPSS software version 20. The impact of assessment days was firstly examined by a one-way ANOVA and no significant difference (*p* = 0.858) was found. Thus, we combined the data for further analysis. A mixed between–within subjects ANOVA was conducted, with six light sources and three types of meat (for the sensory experiment only) as within-subjects factors and experience as the between-subjects factor. If the interaction between two factors was significant, the simple effect of each factor was then analyzed. If the interaction was not significant, the main effects of the factor were interpreted. In these analyses, the degrees of freedom were adjusted by the method of Greenhouse–Geisser [45], and if the sphericity assumption was violated via the Mauchly sphericity test. A probability of *p* = 0.05 was used as the significance level for statistical testing.

For the color discrimination test, after the observer had completed the test, the color discrimination ability of an individual participant was represented by an error score computed from the sorted order and the standard order [46]. The higher the error score, the poorer the color discrimination capability, and vice versa. Such a calculation was automatically implemented with an FM-100 hue test scoring tool provided by X-Rite. These error scores would then be analyzed using IBM SPSS.

## 3. Results

### 3.1. Sensory Evaluation Results

#### 3.1.1. Observer Variability

The intra-observer variability of ratings for visual preference, freshness, and purchase intention were 31, 33, and 39, respectively, with a mean value of 34.3. For inter-observer variability, the STRESS values were 26, 27, and 29, respectively, for the three scales, with a mean of 27.3.

#### 3.1.2. Overall Results

As the Pearson correlation coefficients showed, the participants’ responses to the three scales were highly correlated (all r > 0.972). Principal component analysis of the three groups of ratings yielded a single factor with an eigenvalue > 1, which explained 98.65% of the total variance. This result suggests that these scales tended to measure an underlying structure. The attribute that loaded highest on this factor was the freshness item (0.996). Therefore, we decided to show the analysis only for freshness ratings, as the results for the other scales were highly consistent.

**Figure 6:**
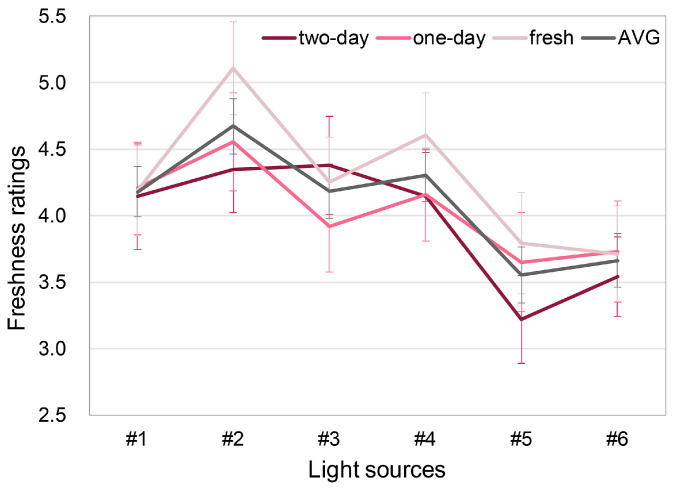
Mean freshness ratings of the three types of pork under six light sources. “fresh”, “one-day”, and “two-day” indicate pork products purchased on the day of the experiment, and purchased one day and two days before the experiment, respectively. The error bars denote 95% confidence intervals (color online only).

Figure 6 shows the mean ratings of the observers for the freshness of the three types of pork under six light sources. Overall, the ratings of the fresh pork display lamps were higher than those of the standard LED lamps, which means that pork products were perceived to be fresher under fresh pork display lamps. Only under fresh pork display lamp #2 and LED white lamp #5 could the observers correctly sort the freshness order of the pork products.

#### 3.1.3. Impact of Lighting on Freshness Evaluation 

Repeated measures ANOVA for the freshness ratings showed a significant main effect for the light source [F (3.772, 230.112) = 15.487, *p* < 0.001, η^2^ = 0.202]. The ratings of the fresh pork display lamps were significantly higher than that of the standard LED lamps, with the ratings for the #2 fresh pork display lamp being the highest. The post hoc test for the significant main effects of the light source revealed that the freshness ratings among these light sources were significantly different: the ratings for standard LED lamps #5 and #6 were significantly lower than those of the four fresh pork display lamps (all Δ > 0.5; Δ: difference value). Among the fresh pork display lamps, the rating for #2 was significantly higher than #1 and #3 (Δ = 0.48, 0.49). That is, participants regarded the pork products seen under fresh pork display lamp #2 as the freshest. 

#### 3.1.4. Impact of Lighting on Freshness Distinction

As can be seen in Figure 6, the fresh pork display lamp #2 and standard LED white lamp #5 enabled observers to accurately distinguish the degree of freshness of pork products, while other lamps could not. A repeated measures ANOVA showed that there was no significant interaction between meat (degree of freshness) and light source [F (8.208, 500.66) = 1.856, *p* = 0.06, η^2^ = 0.03], but the *p* value was very close to the significance level. Thus, it might be meaningful to further analyze the observers’ freshness distinction performance under each light source, as shown in Table 2. 

As shown in Table 2, there was a significant difference in ratings for pork freshness under the fresh pork display lamp #2 and standard LED lamp #5, but such a significant difference only existed between the two-day pork and the fresh pork (Δ = 0.76, *p* = 0.005; Δ = 0.57, *p* = 0.015, respectively). There was no significant difference in the freshness ratings between the one-day pork and the other two types. For other lamps, none of the lamps generated significant differences in pork freshness evaluations.

#### 3.1.5. Impacts of Experience 

**Figure 7:**
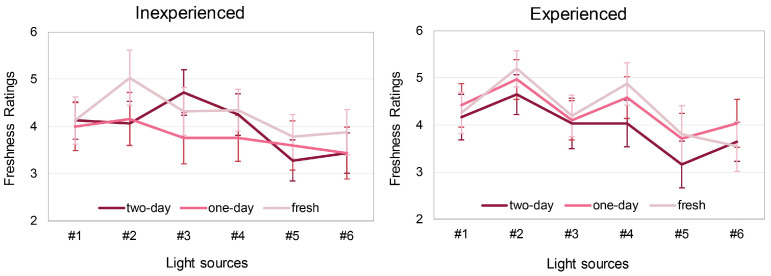
Freshness ratings of experienced and inexperienced observers. The error bars denote 95% confidence intervals (color online only).

For both observer groups, the light source had a significant effect on their evaluation (*p* < 0.001). As shown in Figure 7, for inexperienced observers, the ratings under fresh pork display lamp #2 were significantly higher than under standard LED lamp #6 (Δ = 0.833, *p* = 0.006), but there was no significant difference between ratings for the other lamps. For the experienced group, the ratings under fresh pork display lamp #2 were significantly higher than ratings under fresh pork display lamps #1, #3, #5, and standard LED lamp #6 (Δ = 0.656, *p* = 0.001; Δ = 0.828, *p* = 0.001; Δ = 1.376, *p* < 0.001; Δ = 1.194, *p* < 0.001, respectively). Meanwhile, they also gave significantly higher ratings under fresh pork display lamp #4 than under standard LED lamps #5 and #6 (Δ = 0.935, *p* = 0.013; Δ = 0.753, *p* = 0.019). 

It can also be seen from Figure 7 that the freshness distinction ability of the experienced observers is much stronger than that of the inexperienced observers. As shown in the Figure 7, the inexperienced group could not correctly judge the freshness of the three types of pork while the experienced group performed much better, even though under certain lighting conditions they also made mistakes. According to the results of a repeated measures ANOVA test, the interaction between meat freshness and purchasing experience was significant (*p* = 0.029, η^2^ = 0.057). For the inexperienced group, there was no significant difference between ratings for the three types of meat (*p* = 0.119), while for the experienced group, there was (*p* = 0.01). The experienced observers could distinguish the difference between the fresh pork and the pork products that were purchased 48 h earlier (Δ = 0.366, *p* = 0.031), but they could not distinguish the difference between the one-day pork and the other two types. 

### 3.2. Color Discrimination Results

**Figure 8:**
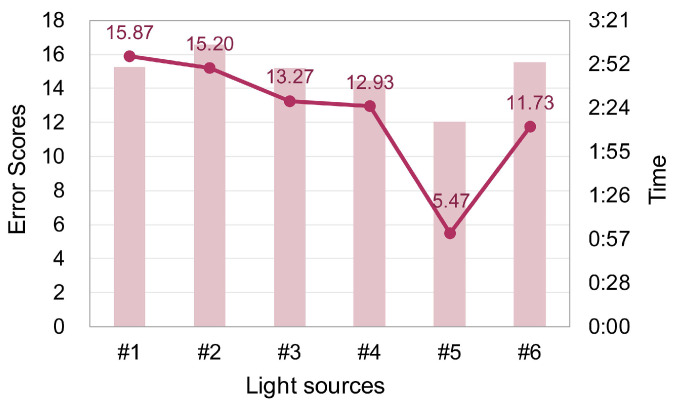
Mean error scores and test time of the color discrimination test under six experimental light sources (color online only).

The results of the color discrimination experiment are shown in Figure 8. As can be seen in Figure 8, the error scores under standard LED lamp #5 were significantly lower than that for other light sources. A repeated measures ANOVA showed that the light source had a significant effect on the color discrimination performance [F (3.504, 98.2) = 5.957, *p* < 0.001, η^2^ = 0.175]. No significant difference [F (1, 28) = 1.444, *p* = 0.24, η^2^ = 0.049] on sex and sex–lighting interaction (*p* = 0.482) was found.

The post hoc test for the significant main effects of the light source showed that the error score under the standard LED lamp #5 was significantly lower than under the other five sources (*p* = 0.001, *p* < 0.001, *p* = 0.005, *p* = 0.004, *p* = 0.019, respectively). No significant difference between fresh pork display lamps and standard LED #6 was found. Figure 8 also illustrates the time used for the color discrimination experiment under each light source, with the shortest time for standard LED lamp #5 and the longest time for fresh pork display lamp #2. Similar to the results on error scores, the light source also had a significant effect [F (3.38, 70.97) = 4.505, *p* = 0.004, η^2^ = 0.177] on test time. The post hoc test for the significant main effects of the light source showed that the time used for each test under the standard LED lamp #5 was significantly shorter than under lamps #2, #3, and #6 (*p* = 0.024, *p* = 0.045, *p* = 0.029, respectively). It seemed that under standard LED lamp #5, people exhibited the best color discrimination performance in both accuracy and efficiency. 

## 4. Discussion

As described in Section 3.1.2 above, the three visual scales of visual preference, freshness, and purchase intention are highly correlated. This finding clearly agrees with our former work regarding the visual effect of lighting on bread and cakes [47], in which we found that human responses of purchase intention, preference, attractiveness, appetite, sweetness, and freshness were closely related. As we speculated, it might not be easy for the participants to clearly differentiate the multi-dimensional and highly correlated judging scales during the visual test. Nevertheless, the consistency among ratings for different scales should undoubtedly represent the subjects’ overall sensory response to the experimental pork products. The STRESS values reported in Section 3.1.1 indicate higher observer variability than our former work on the color rendition of lighting [47,48,49]. Such a result implies that judging the color appearance of pork products under the experimental light sources is a harder task for observers, with a higher degree of uncertainty. Fortunately, the intra-observer variability of the ratings for freshness is 33 and the inter-observer variability is smaller than 30, which could be regarded as acceptable according to Melgosa et al. [44]. Furthermore, note that in this study, the light deterioration issue was not taken into consideration since in each experimental day the pork products with different degrees of freshness were simultaneously displayed under the light sources for only 6–7 h. As pointed out by Kropf [18], the human eyes are not so sensitive as to be able to detect these changes in such a short time.

### 4.1. Effects of Fresh Pork Display Lamp on Freshness Evaluation

As reported in Section 3.1.3, and shown in Figure 6, for meats that are at the same freshness level, the four fresh pork display lamps significantly promoted the freshness assessment when compared to the ordinary LED lamps. This result clearly demonstrates the consumers’ annoyance: in the market, the meat looks fresh and shiny under fresh pork display lamps (e.g., #1–#4), but it appears dim and stale at home under more typical lighting (e.g., #5 and #6). Obviously, the unique SPDs of fresh pork display lamps have played a decisive role.

In 1956, Clark [25] stated that light sources with a closer fit to the reflectance pattern for the meat will bring out the natural appetizing appearance of that product. However, as shown in Figure 1b, the four fresh pork display lamps examined in this study exclusively have two narrow peaks near the wavelengths of 460 nm (blue) and 640 nm (red) and they are not similar to the reflectance of the pork product (Figure 3). We have calculated the Fréchet distance (a distance used to quantify the similarity of curves, see Alt and Godau [50], where a lower value corresponds to a higher degree of similarity) between the spectral reflectance of the pork products and the SPDs of the experimental light sources. It was found that the average distances between the SPDs of lamps #5 and #6 and the pork reflectance (0.69, 0.66, respectively) are smaller than the average distances between the SPDs of #1–#4 and the pork reflectance (0.69–0.79). Such a result contradicts the opinion of Clark [25]. Moreover, according to the previous literature, a lamp spectrum with more red wavelengths could enhance the freshness evaluation of meat product [27,51]. In this study, however, in addition to the red irradiation, all the fresh pork display lamps have strong irradiation of the blue band of the spectrum. From Figure 1b and Figure 6, we can further conclude that the fresh pork display lamps that resulted in the highest freshness ratings (i.e., #2 and #4) both have equal-magnitude emissions in red and blue wavelengths. 

As far as we believe, the partial inconsistency between our results and former conclusions should be ascribed to the fact that in the past studies, researchers did not investigate light sources with such peculiar SPDs. Instead, the light sources they adopted were mainly of a continuous spectrum with much more evenly distributed energy. In addition, in former studies, such as the research of Barbut [27] and Sáenz et al. [51], multiple meat or meat products were used, including beef, pork, chicken, and sausages, so the statement that red light enhances freshness should be wisely considered as a general conclusion. As a matter of fact, in previous Chinese supermarkets, different fresh meat display lamps with different SPDs have been used to illuminate different kinds of meat. Those lamps all possessed a red radiation component, which agrees well with the above statements, but they also had other bands of wavelengths to enhance the sensory evaluation for various meats. As for this work, the pork belly joints consisted of lean meat, fat, and pigskin and they exhibit approximately two colors: red for lean meat and white for fat and pigskin. If it is assumed that the red light should promote the judgment of freshness of the red component of pork, it is likely that the blue emission in the fresh pork display lamp might make the pigskin and fat more appealing. To further validate this opinion, one could repeat our experiment with either lean pork or pure fat. 

### 4.2. Effects of Fresh Pork Display Lamp on Freshness Distinction

The fresh pork display lamps are specialized for promoting the freshness evaluation of the meat product. Obviously, appropriate modification of the meat’s visual appearance should be encouraged. It not only improves the enjoyment of a consumers’ purchasing experience, but also helps to sell the goods that some consumers improperly regard to be of poor quality when merely judging by appearance. For instance, Li and Liu [52] have reported that if fresh beef is distinguished by a red–brown color, it is probably not desired by consumers and is regarded as unattractive or spoiled, even though the meat quality is actually acceptable.

On the other hand, it is unreasonable if the improvement in the meat appearance significantly misleads the consumers and detracts from their ability to distinguish freshness. As shown in Figure 6, the freshness ratings under fresh pork display lamps were significantly higher than that of the ordinary lamps and most of the fresh pork display lamps (#1, #3, #4) in this work masked the freshness of pork products, which echoes the common complaints of current Chinese consumers. In fact, similar findings have also been reported by Hasenbeck et al. [53]. In their work, the authors stated that in red lighting conditions, participants might have more difficulty in obtaining detailed information about bell peppers, since the lighting alters their natural color.

To further explain the results shown in Figure 6, a color appearance analysis was conducted. Three color appearance attributes of the pork colors were calculated using the CIECAM16 model [54], including chroma (C), lightness (J), and hue (H). The CIECAM16 model is a comprehensive model that takes into account the effects of different viewing conditions (illumination, environment, background, visual adaptation, etc.) on the appearance of an object color. Table 3 summarizes the correlation between the average freshness ratings of observers under each experimental light source and the corresponding color appearance values of pork colors. The freshness evaluation of observers is mainly correlated to the chroma (r = 0.83) and lightness (r = 0.71) of pork products.

**Figure 9:**
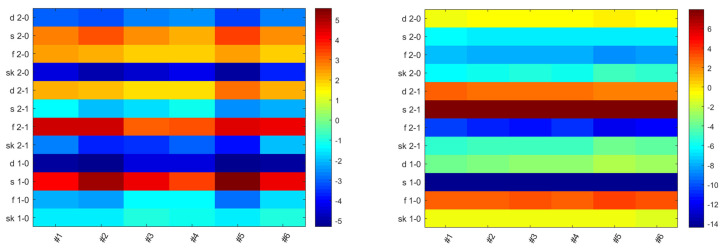
Chroma (**left**) and lightness (**right**) difference between pork products with different degrees of freshness under the six experimental light sources. The abscissa refers to the six experimental light sources and the ordinate represents the difference in color appearance attributes. For instance, “d 1−0” in chroma means the chroma difference between one-day and fresh pork in deep portion of lean meat. The magnitude of the difference is indicated by color (color online only).

Thus, we calculated the difference in chroma and lightness of the three types of meat under six experimental lamps. As shown in Figure 9, we found that under light sources #2 and #5, the chroma difference between the pork products of different degrees of freshness was more obvious, but there were no similar results for lightness difference. Such a finding provides a possible explanation for the freshness distinction results described in Section 3.1.4: light sources #2 and #5 helped observers to correctly judge the freshness of meat by maximizing the chroma difference between pork products with different degrees of freshness. Similarly, light sources #1, #3, and #4 impaired the freshness distinction ability of buyers since their color rendition property masked the chroma difference. 

In addition, according to Section 3.1.4, we found a significant difference only existed between the two-day pork and the fresh pork. However, this result could not be perfectly explained by Figure 9, which shows that the chroma difference between the two-day pork and fresh pork is smaller than that between the one-day pork and the other two types. According to a post-experiment interview after the sensory evaluation experiment, many experienced participants agreed that color was the main factor in judging freshness, but some also mentioned that they judged the freshness of meat to some extent based on its gloss and surface moisture. This was consistent with the findings of Fortomaris et al. [55], who reported that consumers most often used two of the four characteristics, including color, fat cover, marbling, and drip, to make their selection. Thus, we suspect that when evaluating the freshness of pork, experienced buyers might simultaneously take color and surface moisture into account. 

### 4.3. Effects of Purchase Experience on Freshness Distinction

As described in Section 3.1.5, the observers with no experience of buying meat could not distinguish the freshness of the pork products while the experienced observers could. As can be seen from Figure 7, in general, the rank orders of freshness evaluation for the three groups of meat for experienced observers are much more stable than the inexperienced observers. This should be attributed to the impact of familiarity on freshness judgment. In 2002, O’Sullivan et al. [56] stated that trained sensory panelists were able to differentiate four experimental meat groups and they were more effective in evaluating the color quality of samples. Furthermore, O’Sullivan et al. [57] showed that the sensory visual assessment of meat products can be undertaken effectively without training when the product, or rather the color of that product, is familiar to the assessors. In studies on color perception, it is also widely acknowledged that people do not very much mind about the original color of objects with which they are unfamiliar, but they have precise ideas about what the colors of familiar objects should be [58,59,60]. 

Thus, based on the above discussions, we conclude that under fresh pork display lamps, both experienced and inexperienced observers were likely to overestimate the freshness of the illuminated pork. Meanwhile, those lamps with improper spectral compositions which have particularly high but unequal emission peaks in the red and blue wavelengths might to some extent impair the freshness distinction ability of experienced observers. Inexperienced observers, however, were immune to this negative influence since intrinsically they do not possess that ability.

### 4.4. Analysis on Color Discrimination

The color discrimination test served as the supplementary experiment for the above sensory evaluation experiment. By this test, we wanted to justify the opinion that the color discrimination of illumination is reduced because of the falsely saturated colors [30]. In addition, we wanted to investigate whether such impairment, if it exists, leads to the lowering in the ability of an observer to distinguish freshness. Note that in this test, only observers with purchasing experience of pork products were recruited. Inexperienced observers were excluded due to the lack of freshness distinction ability, as demonstrated above. 

As depicted in Figure 8, the standard LED lamp #5 provided the best color discrimination performance in both accuracy and efficiency while the color discrimination capability of the four fresh pork display lamps was relatively poor. Such a finding consolidates the statement that color discrimination ability is impaired by the over-saturated light sources. Similar findings were also reported by Boissard and Fontoynont [61], where the authors pointed out that the light emitted by a halogen lamp was very reddish and this did not permit an observer to be able to distinguish the adjacent color of a color chart and a painting. Apparently, the over-saturated effect of fresh pork display lamps is much stronger than a halogen lamp. 

Table 1 shows the values of our newly proposed color discrimination metric (CDM) [36] for the six experimental light sources. Such a measure is a state-of-art metric for quantifying the color discrimination capability of lighting and its superiority over 29 typical color quality metrics has been validated based on a meta-analysis of 16 groups of visual data. As shown in Table 1, the CDM value of lamp #5 is much higher than that of the other light sources. For the four fresh pork display lamps, their CDM values are exclusively below zero, which is extremely rare among ordinary white light sources.

Our original hypothesis was that inferior color discrimination capability leads to poor performance in freshness distinction. According to the results shown in Figure 6 and Figure 8, it was found that lamps #1, #3, and #4 supported this opinion but lamp #2 did not. That is, although people performed poorly in the color discrimination test under fresh pork display lamp #2, generally they could distinguish different freshness levels under that light. Such a finding implies that the color discrimination process examined in the FM-100 hue test might not necessarily be in close relation with freshness distinction. A possible explanation for such results is that in FM-100 hue test, only the discrimination of *hue* is examined, while according to the discussion in Section 4.2, the visual attribute that impacts freshness distinction is *chroma* difference. Thus, it is possible that when consumers complain that they cannot clearly discriminate colors under fresh pork display lamps and thus lose the ability to distinguish freshness, they essentially mean that they could not differentiate the chroma difference in the meat rather than the difference in hue, under that lighting.

## 5. Conclusions

In this study, two sensory experiments were carried out to investigate the impacts of fresh pork display lamps on the sensory assessment for pork products. It is clearly demonstrated that the fresh pork display lamps exaggerated the freshness evaluation for pork products and misled consumers. Meanwhile, fresh pork display lamps with an inappropriate design of the radiation spectrum which have particularly high but unequal emission peaks in the red and blue wavelengths also seriously impaired the freshness distinction ability of consumers. This problem is mainly related to experienced participants with pork purchasing experience since inexperienced participants intrinsically do not possess that ability. Meanwhile, the results of color appearance analysis and FM-100 hue test mutually demonstrated that it is chroma difference rather than hue difference that was closely correlated to the freshness distinction of pork product. In this study, the authors have provided scientific evidence of why the usage of fresh pork display lamps with unreasonable spectrum structures should be banned and would like to remind lamp users and manufacturers to not only be concerned about the improvement in the color appearance of pork, but also the need for consumers to be aware of the authentic quality of the pork products.

## Figures and Tables

**Table 1:** The colorimetric properties of the experimental light sources and the corresponding values of typical color quality metrics.

ID	#1	#2	#3	#4	#5	#6
CCT (k)	1662	>100,000	>100,000	2248	5356	2861
x	0.379	0.313	0.298	0.343	0.336	0.448
y	0.187	0.166	0.167	0.183	0.350	0.409
Duv	−0.111	−0.112	−0.098	−0.115	0.003	0.001
CRI	−38	−62	−18	−7	98	98
GAI	165	170	160	156	93	54
CDM	−3.08	−2.80	−1.40	−1.96	5.55	1.33
MCPI	86.56	--	--	103.62	112.49	90.73

(x, y): CIE 1931 chromaticity coordinates; Duv [33]: the distance from the test chromaticity coordinates to the Planckian locus; CRI [34]: CIE general color rendering index; GAI [35]: gamut area index; CDM [36]: color discrimination metric; MCPI [37]: color preference index based on meta-analysis.

**Table 2:** Results of the post hoc test regarding observers’ freshness distinction performance under six light sources.

ID	Sum of Squares	df	Mean Square	F	Sig.
#1	0.138	2	0.069	0.049	0.952
#2	19.566	2	9.783	5.405	0.006
#3	7.122	2	3.561	2.087	0.128
#4	8.603	2	4.302	2.580	0.080
#5	11.143	2	5.571	4.481	0.013
#6	1.407	2	0.704	0.382	0.683

df: degree of freedom; Sig.: significance (*p* value).

**Table 3:** Pearson correlation coefficients between color appearance attributes and subjective freshness ratings under different experimental light sources.

Attributes	Type	d	s	f	sk	AVG
C	two-day pork	0.73	0.90	0.92	0.90	0.83
	one-day pork	0.89	0.92	0.86	0.88	
	fresh pork	0.71	0.77	0.76	0.77	
H	two-day pork	−0.18	0.31	0.92	0.60	0.39
	one-day pork	0.16	−0.27	0.80	0.80	
	fresh pork	0.23	−0.20	0.77	0.77	
J	two-day pork	0.79	0.83	0.80	0.73	0.71
	one-day pork	0.80	0.79	0.72	0.75	
	fresh pork	0.60	0.62	0.55	0.55	

Two-day pork: pork products purchased two days before the experiment; one-day pork: pork products purchased one day before the experiment; fresh pork: pork products purchased on the day of the experiment; d: deep portion of the pork (darker); s: the superficial portion (paler); f: fat; sk: pigskin; C: chroma; H: hue; J: lightness.

## Data Availability

The original contributions presented in this study are included in the article; further inquiries can be directed to the corresponding author.

## References

[1] OECD/FAO (2020). OECD-FAO Agricultural Outlook.

[2] World Health Organization (2021). Estimating the Burden of Foodborne Diseases: A Practical Handbook for Countries. https://www.who.int/publications/i/item/9789240012264.

[3] Blasco J., Aleixos N., Moltó E. (2003). Machine Vision System for Automatic Quality Grading of Fruit. Biosyst. Eng..

[4] Pedreschi R., Hertog M., Lilley K.S., Nicolaï B. (2010). Proteomics for the Food Industry: Opportunities and Challenges. Crit. Rev. Food Sci. Nutr..

[5] Cubero S., Aleixos N., Moltó E., Gómez-Sanchis J., Blasco J. (2010). Advances in Machine Vision Applications for Automatic Inspection and Quality Evaluation of Fruits and Vegetables. Food Bioprocess Technol..

[6] Foroni F., Pergola G., Rumiati R.I. (2016). Food color is in the eye of the beholder: The role of human trichromatic vision in food evaluation. Sci. Rep..

[7] Sharif M., Butt M., Sharif H., Nasir M. (2017). Sensory Evaluation and Consumer Acceptability. Handbook of Food Science and Technology.

[8] Polkinghorne R., Philpott J., Watson R., Tarr G. (2017). Impacts on Consumer Acceptance of Beef from Interactions between pH, Meat Colour and Packaging.

[9] Nauman H.D., McBee J.L., Brady D.E. (1957). Colour stability of frozen beef as influenced by illumination, temperature and storage. Food Technol..

[10] Pangborn R.M., Kare M.R., Maller O. (1967). Some aspects of chemorecept in human nutrition. The Chemical Senses and Nutrition.

[11] Smith G.C., Belk K.E., Sofos J.N., Tatum J.D., Williams S.N., Decker E., Faustman C., Lopezbote C.J., Decker E.A., Faustman C., Lopez-Bote C.J. (2000). Economic implications of improved colour stability in beef. Antioxidants in Muscle Foods: Nutritional Strategies to Improve Quality.

[12] Jantathai S., Danner L., Joechl M., Dürrschmid K. (2013). Gazing behavior, choice and color of food: Does gazing behavior predict choice?. Food Res. Int..

[13] Quevedo R., Valencia E., Cuevas G., Ronceros B., Pedreschi F., Bastías J.M. (2013). Color changes in the surface of fresh cut meat: A fractal kinetic application. Food Res. Int..

[14] Passetti R.A.C., Resconi V.C., Çakmakçı C., del Mar Campo M., Kirinus J.K., Passetti L.C.G., Guerrero A., do Prado I.N., Sañudo C. (2019). Number of consumers and days of display necessary for the assessment of meat colour acceptability. Food Res. Int..

[15] Martínez L., Cilla I., Antonio J., Roncalés P. (2007). Effect of illumination on the display life of fresh pork sausages packaged in modified atmosphere. Influence of the addition of rosemary, ascorbic acid and black pepper. Meat Sci..

[16] Mancini R.A., Hunt M.C. (2005). Current research in meat color. Meat Sci..

[17] Hunt M.C. Meat colour measurements. Proceedings of the Reciprocal Meat Conference.

[18] Kropf D.H. Effects of retail display conditions on meat colour. Proceedings of the Reciprocal Meat Conference.

[19] Barbut S. (2001). Acceptance of fresh chicken meat presented under three light sources. Poult. Sci..

[20] Kim M.J., Parvin R., Mushtaq M.M.H., Hwangbo J., Kim J.H., Na J.C., Kim D.W., Kang H.K., Kim C.D., Cho K.O. (2013). Influence of monochromatic light on quality traits, nutritional, fatty acid, and amino acid profiles of broiler chicken meat. Poult. Sci..

[21] Corrêa T.Q., Blanco K.C., Garcia É.B., Lara Perez S.M., Chianfrone D.J., Morais V.S., Bagnato V.S. (2020). Effects of ultraviolet light and curcumin-mediated photodynamic inactivation on microbiological food safety: A study in meat and fruit. Photodiagn. Photodyn. Ther..

[22] Soro A.B., Harrison S.M., Whyte P., Bolton D.J., Tiwari B.K. (2022). Impact of ultraviolet light and cold plasma on fatty acid profile of raw chicken and pork meat. J. Food Compos. Anal..

[23] Djenane D., Sánchez-Escalante A., Beltrán J.A., Roncalés P. (2001). Extension of the retail display life of fresh beef packaged in modified atmosphere by varying lighting conditions. J. Food Sci..

[24] Steele K.S., Weber M.J., Boyle E.A.E., Hunt M.C., Lobaton-Sulabo A.S., Cundith C., Hiebert Y.H., Abrolat K.A., Attey J.M., Clark S.D. (2016). Shelf life of fresh meat products under LED or fluorescent lighting. Meat Sci..

[25] Clark C.N. (1956). The basis for appropriate lighting for meat displays. Proceedings of the Meat Industry Research, American Meat Institute.

[26] Hunt M.C., Smith R.A., Kropf D.H., Tuma H.J. (1975). Factors affecting showcase colour stability of frozen lamb in transparent film. J. Food Sci..

[27] Barbut S. (2001). Effect of illumination source on the appearance of fresh meat. Meat Sci..

[28] Barbut S. (2005). Effect of enhanced fluorescent light on acceptability of meat cuts. J. Muscle Foods.

[29] Smet K.A.G., Roelandts I., Teunissen K., Poort S., Hanselaer P. (2018). Application specific extension of the MCRI: Memory colors and preferred colors of reddish meat products. Color Res. Appl..

[30] Mahler E., Ezrati J.J., Viénot F. (2009). Testing LED lighting for colour discrimination and colour rendering. Color Res. Appl..

[31] Farnsworth D. (1943). The Farnsworth-Munsell 100-hue and dichotomous tests for color vision. J. Opt. Soc. Am..

[32] Wang B., Shen C., Cai Y., Liu D.Y., Gai S. (2011). The purchase willingness of consumers for red meat in China. Meat Sci..

[33] Ohno Y. (2014). Practical Use and Calculation of CCT and Duv. Leukos.

[34] Nickerson D., Jerome C.W. (1965). Color rendering of light sources: CIE method of specification and its application. Illum. Eng..

[35] Freyssinier J.P., Rea M. A two-metric proposal to specify the color-rendering properties of light sources for retail lighting. Proceedings of the Tenth International Conference on Solid State Lighting.

[36] Liu Q., Liu Y., Pointer M.R., Huang Z., Wu X.W., Chen Z.Y., Luo M.R. (2020). A colour discrimination metric based on the neutrality of lighting and hue transposition quantification. Opt. Lett..

[37] Huang Z., Chen W., Liu Q., Wang Y., Pointer M.R., Liu Y., Liang J.X. (2021). Towards an optimum colour preference metric for white light sources: A comprehensive investigation based on empirical data. Opt. Express.

[38] (2017). Method for Analysis of Hygienic Standard of Food.

[39] Dave D., Ghaly A.E. (2011). Meat spoilage mechanisms and preservation techniques: A critical review. Am. J. Agric. Biol. Sci..

[40] (2017). Fresh and Frozen Livestock and Poultry Products.

[41] Liang J.X., Wan X.X. (2017). Optimized method for spectral reflectance reconstruction from camera responses. Opt. Express.

[42] Thornton W.A. (1972). Color-Discrimination Index. J. Opt. Soc. Am. A.

[43] Luo M.R. (2011). The quality of light sources. Color Technol..

[44] Melgosa M., García P.A., Gómez-Robledo L., Shamey R., Hinks D., Cui G., Luo M. (2011). Notes on the application of the standardized residual sum of squares index for the assessment of intra-and inter-observer variability in colour-difference experiments. J. Opt. Soc. Am. A.

[45] Greenhouse S.W., Geisser S. (1959). On methods in the analysis of profile data. Psychometrika.

[46] Huang Z., Liu Q., Liu Y., Pointer M.R., Luo M.R., Wang Q., Wu B. (2019). Best lighting for jeans, part 1: Optimizing color preference and color discrimination with multiple correlated color temperatures. Light. Res. Technol..

[47] Chen W., Wu X.W., Liu Z.Y., Liu Y., Liu Q., Pointer M.R., Liang J.X., Khanh T.Q. (2022). The impact of illuminance level, correlated colour temperature and viewing background on the purchase intention for bread and cakes. Food Qual. Prefer..

[48] Huang Z., Liu Q., Luo M.R., Pointer M.R., Liu Y., Wang Y., Wu X.W. (2021). Whiteness and preference perception of white light sources: A case study at 5500 K with positive and negative Duv values. Optik.

[49] Deng X., Liu Y.X., Tian B.L., Zhang W., Yu F., Liu Q. (2022). Experimental setting and protocol impact human colour preference assessment under multiple white light sources. Front. Neurosci..

[50] Alt H., Godau M. (1995). Computing the Fréchet distance between two polygonal curves. Int. J. Comput. Geom. Appl..

[51] Sáenz C., Hernández B., Beriain M.J., Lizaso G. (2005). Meat colour in retail displays with fluorescent illumination. Color. Res. Appl..

[52] Li Y.F., Liu S.M. (2012). Reducing lipid peroxidation for improving colour stability of beef and lamb: On-farm considerations. J. Sci. Food Agric..

[53] Hasenbeck A., Cho S., Meullenet J.F., Tokar T., Yang F., Huddleston E.A., Seo H.S. (2014). Colour and illuminance level of lighting can modulate willingness to eat bell peppers. J. Sci. Food Agric..

[54] Li C.J., Li Z.Q., Wang Z.F., Xu Y., Luo M.R., Cui G.H., Melgosa M., Brill M.H., Pointer M. (2017). Comprehensive colour solutions: CAM16, CAT16, and CAM16-UCS. Color Res. Appl..

[55] Fortomaris P., Arsenos G., Georgiadis M., Banos G., Stamataris C., Zygoyiannis D. (2006). Effect of meat appearance on consumer preferences for pork chops in Greece and Cyprus. Meat Sci..

[56] O’Sullivan M.G., Byrne D.V., Stagsted J., Andersen H.J., Martens M. (2002). Sensory colour assessment of fresh meat from pigs supplemented with iron and vitamin E. Meat Sci..

[57] O’Sullivan M.G., Byrne D.V., Martens M. (2003). Evaluation of pork meat colour: Sensory colour assessment using trained and untrained sensory panellists. Meat Sci..

[58] Yendrikhovskij S.N., Blommaert F.J.J., de Ridder H. (1999). Color reproduction and the naturalness constraint. Color Res. Appl..

[59] Boust C., Brettel H., Viénot F., Alquié G., Berche S. (2006). Color Enhancement of Digital Images by Experts and Preference Judgments by Observers. J. Imaging Sci. Technol..

[60] Huang Z., Liu Q., Westland S., Pointer M.R., Luo M.R., Xiao K.D. (2017). Light dominates colour preference when correlated colour temperature differs. Lighting Res. Technol..

[61] Boissard S., Fontoynont M. (2009). Optimization of LED-based light blendings for object presentation. Color Res. Appl..

